# Design and Systematic Evaluation of a Multi-Layered Mattress System for Accurate, Unobtrusive Capacitive ECG Monitoring

**DOI:** 10.3390/bioengineering12121348

**Published:** 2025-12-10

**Authors:** Rui Cui, Kaichen Wang, Xiongwen Zheng, Jiayi Li, Siheng Cao, Hongyu Chen, Wei Chen, Chen Chen, Jingchun Luo

**Affiliations:** 1 Human Phenome Institute, Fudan University, Shanghai 200032, China; rcui22@m.fudan.edu.cn (R.C.); 23112030013@m.fudan.edu.cn (J.L.); shcao24@m.fudan.edu.cn (S.C.); 2 School of Information Science and Technology, Fudan University, Shanghai 200032, China; 23110720140@m.fudan.edu.cn (K.W.); 22210720323@m.fudan.edu.cn (X.Z.); 3 Greater Bay Area Institute of Precision Medicine, Guangzhou 511458, China; chenhongyudesign@outlook.com; 4 School of Biomedical Engineering, The University of Sydney, Darlington, NSW 2006, Australia; wei.chenbme@sydney.edu.au

**Keywords:** capacitive ECG, flexible sensor, active electrodes, adaptive fourier decomposition

## Abstract

Capacitive ECG (cECG) technology offers significant potential for improving comfort and unobtrusiveness in long-term cardiovascular monitoring. Nevertheless, current research predominantly emphasizes basic heart rate monitoring by detecting only the R-wave, thereby restricting its clinical applicability. In this study, we proposed an advanced cECG mattress system and conducted a systematic evaluation. To enhance user comfort and achieve more accurate cECG morphological features, we developed a multi-layered cECG mattress incorporating flexible fabric active electrodes, signal acquisition circuits, and specialized signal processing algorithms. We conducted experimental validation to evaluate the performance of the proposed system. The system exhibited robust performance across various sleeping positions (supine, right lateral, left lateral and prone), achieving a high average true positive rate (TPR) of 0.99, ensuring reliable waveform detection. The mean absolute error (MAE) remains low at 1.12 ms for the R wave, 7.89 ms for the P wave, and 7.88 ms for the T wave, indicating accurate morphological feature extraction. Additionally, the system maintains a low MAE of 0.89 ms for the RR interval, 7.77 ms for the PR interval, and 7.85 ms for the RT interval, further underscoring its reliability in interval measurements. Compared with medical-grade devices, the signal quality obtained by the cECG mattress system is sufficient to accurately identify the crucial waveform morphology and interval durations. Moreover, the user experience evaluation and durability test demonstrated that the mattress system performed reliably and comfortably. This study provides essential information and establishes a foundation for the clinical application of cECG technology in future sleep monitoring research.

## 1. Introduction

Electrocardiograph (ECG) monitoring is one of the most commonly used tests in hospital settings. ECG monitoring plays a vital role in cardiac health management and cardiovascular disease diagnosis to improve patients’ health status and prevent more severe complications, especially when employed as continuous surveillance [1]. Clinical long-term ECG monitoring systems, known as Holter, utilize traditional gel electrodes in contact with the skin to provide high quality ECG monitoring, making them suitable for daytime monitoring. However, Holter monitors have some inherent limitations [2], including the complicated setup, requiring a rigid data acquisition module, long tangling cables, adhesive electrodes, tapes, and a strap. Consequently, these devices often compromise user experience, restrict natural movement, and cause considerable discomfort, particularly by disrupting sleep patterns during nighttime [3]. In addition, skin intolerance of the adhesives required for medical wearable monitoring is another limitation of current devices [4]. This consideration is particularly important for vulnerable populations like the elderly and infants, as their fragile skin is more susceptible to severe complications from infections and wounds. The factors mentioned above would affect compliance with the use of continuous health monitoring devices. Therefore, the development of more unobtrusive and comfortable solutions for nighttime ECG monitoring remains an urgent challenge.

Fortunately, unobtrusive health monitoring offers the potential to overcome the challenge of actively modifying human behavior, paving the way toward precision health [5]. Among this, capacitive ECG (cECG) is a form of unobtrusive health monitoring technology [6,7]. Unlike traditional skin-contact ECG, capacitive ECG technology operates without the need for direct skin contact [8]. Instead, it detects the potential changes of the body’s surface electric field through clothing or hair to record cardiac signals [9]. Compared to silver/silver chloride (Ag/AgCl) electrodes, cECG eliminates issues such as skin discomfort and allergies while minimizing disruptions to daily life and sleep [10]. This contactless approach significantly enhances monitoring comfort, making it well-suited for long-term and nighttime use. Moreover, it has the potential to transform current monitoring methodologies by enabling the early detection of cardiac abnormalities in everyday settings.

Although the signal quality of cECG is less stable compared to contact-based devices, advancements in hardware circuit design [11,12,13,14] and signal processing algorithms [15,16,17] are expected to progressively address this limitation. Such developments position cECG as a promising solution for unobtrusive nighttime cardiac monitoring. In recent years, significant progress was made in the development of cECG systems for nighttime ECG monitoring. Researchers have increasingly focused on designing cECG sensors specifically tailored for integration into beds [18,19,20,21,22,23,24,25,26], making them more practical for nighttime use. Lim et al. [18], Nakamura et al. [19], and Yu et al. [20] used capacitive coupling electrodes integrated into beds with printed circuit boards (PCB) for cECG monitoring. However, the rigid nature of the PCB results in relatively poor comfort. Wang et al. [21] proposed a cECG system based on flexible printed circuit board (FPC). Compared to PCB, FPC is softer and more flexible, offering improved adaptability to the human body. However, prolonged and repeated use of FPC electrodes can result in irreversible deformation. Peng et al. [22] and Takano et al. [23] used conductive fabric as capacitive electrodes for sleep monitoring, which improved comfort significantly. However, flexible fabrics are more susceptible to movements, and when combined with inherent signal attenuation and environmental noise interference, the signal quality remains suboptimal, particularly across varying sleeping positions. To further improve signal acquisition quality with the fabric electrodes, Wang et al. [24] utilized additional parallel capacitors in the pillow. The positive pole of the capacitor is connected to the human epidermis, while the negative pole is connected to the fabric electrode, enhancing the signal-to-noise ratio in the supine position. Although Wang et al. [24] implemented a capacitive ECG configuration, the inclusion of additional capacitors in their design results in partial contact with the body. Furthermore, due to the limited coverage area of the positive pole of the capacitors on the pillow, the proposed system exhibits poor cECG signal quality when the subject is in other sleep position. Xiao et al. [25] and Feng et al. [26] integrated water-absorbing materials with fabric electrodes, ensuring both comfort and improved cECG signal quality. However, the structure of water-absorbing materials changes with repeated use, leading to increased moisture loss and reduced water retention capacity, ultimately causing a decline in performance. Current systems still struggle to achieve long-term comfort and stable signal acquisition. Hard electrode–skin interface materials can cause discomfort, while electrodes incorporating flexible hydrogel layers may suffer from performance degradation over time. As a result, existing cECG mattress systems continue to face substantial challenges in extracting detailed waveform components across different sleep postures, limiting their ability to support reliable morphological analysis. These limitations hinder their potential for broader clinical and home-health applications. In addition, most systems lack integrated evaluation frameworks that combine objective electrophysiological validation, subjective comfort assessment, and durability testing. Collectively, these shortcomings underscore the need for a comprehensive and systematic solution to improve the practicality, robustness, and clinical relevance of unobtrusive cardiac monitoring technologies. Therefore, priority should be given to optimizing electrode design, refining signal processing algorithms, and enhancing device comfort to improve overall robustness and enable consistent acquisition of clean waveforms [27]. In this study, we proposed a cECG system based on flexible active electrodes with an adaptive Fourier Decomposition filtering algorithm. Compared with existing cECG bedding systems [18,19,20,21,22,23,24,25,26], our approach integrates a multi-layered mattress architecture with silver fabric and flexible PCB circuitry, systematically optimized to enhance signal acquisition, reduce common-mode interference, and maximize user comfort. Signal processing algorithms further enable accurate identification and analysis of detailed ECG waveforms across four sleep postures. We also present a systematic evaluation framework that integrates simultaneous Holter validation, assessments of user experience, and industrial-grade durability testing. This holistic evaluation dimension that has not been addressed in prior capacitive ECG studies [11,12,13,14,18,19,20,21,22,23,24,25,26]. Together, these elements distinguish our system from prior cECG platforms and underscore its advancements in design robustness, practical usability, and physiological data quality. Whereas previous systems typically focus on proof-of-concept signal acquisition, our work offers a systematic evaluation of a full cECG system, presenting a more complete and deployable monitoring framework. The holistic evaluation demonstrated that our mattress system achieves performance comparable to traditional contact-based devices while ensuring comfortable and unobtrusive monitoring. Furthermore, the mattress passed durability test in accordance with international standards, confirming its suitability for long-term use. Elderly individuals, people requiring long-term cardiac surveillance, those with chronic cardiovascular conditions, and users who experience discomfort or skin irritation from adhesive electrodes represent key populations that could benefit substantially from this technology. The proposed mattress system is suited for home environments, long-term care facilities, and hospital beds, where unobtrusive and passive data collection can enhance user comfort while providing clinicians with continuous physiological information. By addressing the needs of these specific groups, the system offers meaningful potential to improve clinical monitoring and outcomes.

## 2. Materials and Methods

### 2.1. System Design Overview

**Figure 1:**
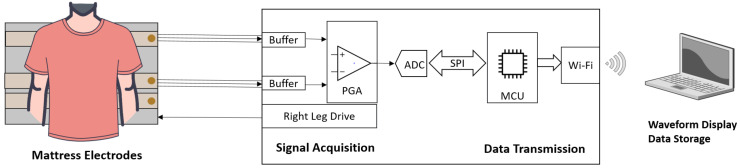
Diagram of capacitive ECG mattress system.

The system comprises three main components: the mattress electrodes unit, the signal acquisition and data transmission unit, and the waveform display and storage unit, as shown in Figure 1. The system operates through cotton sleepwear without direct electrical contact between the electrodes and the skin. These garments act as dielectric layers. The electrode unit consists of active electrodes based on flexible silver fabric, designed in the form of mattress. These flexible active electrodes exhibit excellent electrical performance and mechanical deformation capability, enabling effective mechanical contact and recording of bioelectric signals. The function of the signal acquisition and data transmission unit is to collect analog signals, perform analog computations, and subsequently convert them into digital signals. The acquisition circuitry performs two separate analog computations: a differential computation between the two active electrodes to extract the cECG signal, and a mean computation used by the right leg drive (RLD) circuit. The RLD stage calculates the average potential of the two active inputs, generates a negatively amplified version of this mean signal, and feeds it back to the body to reduce common-mode noise. Utilizing WiFi wireless network technology, the processed digital signals are transmitted to the data display and storage unit. This transmission method facilitates rapid and convenient data transfer to remote devices or cloud servers for further analysis and processing. The data display and storage unit are responsible for processing, displaying, and storing the data. It presents the cECG data in a comprehensible format to the user and can store the data internally or on external storage media for future retrieval and analysis.

### 2.2. Flexible Fabric Active Electrodes

**Figure 2:**
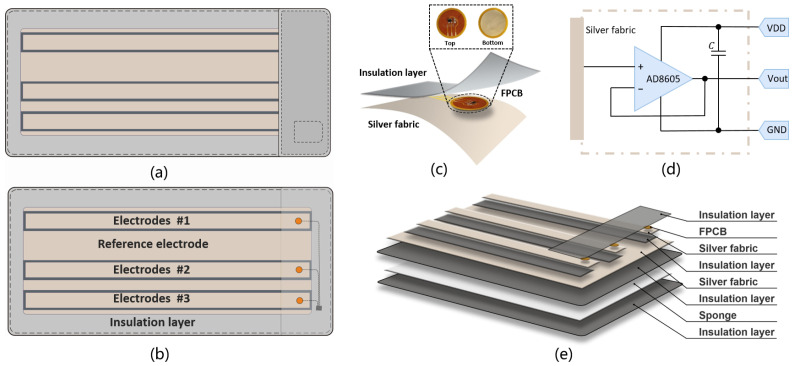
Multi-layered flexible fabric capacitive ECG mattress. (**a**) Overall appearance. (**b**) Electrode arrangement. (**c**) Single capacitive coupled active electrode Structure. (**d**) FPCB circuit diagram. (**e**) Multi-layer electrode structure diagram.

The multi-layered flexible fabric active electrodes comprises three capacitive coupled active electrodes and one reference electrode, as shown in Figure 2. These electrodes are designed in elongated shapes to ensure continuous contact with the body during movements and position changes during nighttime sleep, ensuring stable acquisition of cECG signals without constraint. The elongated capacitive coupled electrodes, labeled #1, #2, and #3, are insulated from each other and share a common reference electrode. Given variations in individuals’ heights and the distance between their ribs and shoulders, electrode #1 is positioned near the shoulder while either electrode #2 or #3 is placed near the rib. Electrodes #1 and #2 form cECG channel 1 (C1), whereas electrodes #1 and #3 constitute cECG channel 2 (C2). Our configuration is designed to be close to the clinical standard II lead. This arrangement provides two redundant cECG channels during sleep, accommodating subjects of different heights. The reference electrode, comprising a large piece of silver fabric, is positioned beneath the three capacitive coupled electrodes. It is connected to the right leg drive circuitry of the signal acquisition module to enhance common mode rejection ratio (CMRR) and mitigate electromagnetic interference. This design ensures accurate and reliable cECG signal acquisition, even in dynamic and varied sleeping positions.

Each capacitive coupled active electrode consists of three layers, namely silver fabric, flexible PCB circuitry and insulation layer, as shown in Figure 2c.

Silver fabric layer serves as the sensing material that comes into contact with the clothes and the body to detect bioelectric signals. Silver fabric is selected due to its excellent electrical properties and biocompatibility. Additionally, the silver fabric is soft, flexible and permeable, making it ideal for continuous and comfortable contact with the body. Using the technique of vacuum sputtering, silver ions are incorporated into textile fibers to produce the silver fabric. Due to the excellent electrical properties, silver fabric efficiently collects and transmits bioelectrical signals. Compared to other metals, silver exhibits superior biocompatibility, minimizing the likelihood of allergic reactions or adverse effects on the human body. Thus, silver fabric preserves the softness characteristic of textiles while offering the electrical properties of metal materials. In contrast to conventional metal electrodes, silver fabric based electrodes demonstrates greater flexibility, making it more suitable for conformal contact with the human body. Additionally, silver fabric exhibits excellent permeable, which can mitigate skin moisture and discomfort, rendering it particularly suitable for prolonged use.

**Figure 3:**
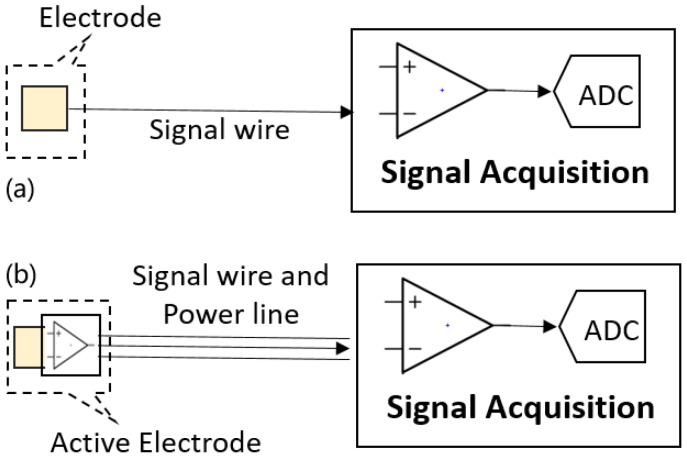
Electrodes integrations in bio-potential recording devices. (**a**) Passive electrodes. (**b**) Active electrodes.

Passive electrodes (Figure 3a) capture raw bioelectric signals, which are then directly transmitted to the signal acquisition system through wires. Due to the high impedance between the body and the electrodes compared to wet electrodes, passive electrodes are more susceptible to external interference [28,29]. Relative motion of the electrode and human body generates significant signals known as motion artifacts. The connecting wires between the electrodes and the front-end amplifier can parasitically act as antennas and capture external electromagnetic interference. Additionally, the wires are more likely to be coupled with the power line, resulting in substantial interference at 50/60 Hz.

An effective method to mitigate these effects is the use of sensors based on active electrodes [30]. As shown in Figure 3b, active electrodes are the electrodes embedded with amplifiers at the electrode side [31], which locally buffer the weak bio-potential signals before transmitting through the cable. Therefore, the sensitivity of the system to noise is minimized by the short distance between the electrode and the signal acquisition system [32].

A flexible PCB circuitry of voltage follower is embedded within the fabric to increase the input impedance and to drive the wires. The voltage follower is one of popular architecture for AEs due to its balanced analog performance and relatively simple structure, offering high input impedance, low output impedance, and minimal gain variation [33]. The chosen operational amplifiers are AD8605 (Analog Devices Inc., Wilmington, MA, USA), which feature low offset (maximum 65 μV), low noise (8 nV/$\sqrt{\mathrm{Hz}}$), and low input bias currents (maximum 1 pA). Additionally, the compact SOT23 package is ideal for integration within the AE design. A 0603 100 nF capacitor is installed for power supply decoupling.

Compared to the boosted input impedance provided by the active electrodes, fluctuations of the electrode–skin interface impedance are relatively minimized, thereby reducing motion artifacts [34]. The buffers on the electrode side also drive the wires, helping to eliminate power line interference [35].

The insulation layer is made from high resistance fabric, in contrast to the silver fabric. This layer aims to ensure electrical isolation between the three capacitive coupled electrodes and the reference electrode. The high resistance fabric exhibits insulating properties, blocking the conduction of signals to prevent unwanted interference. By maintaining robust electrical isolation, the insulation layer ensures the independence of signals between each electrode, thereby enhancing measurement accuracy and reliability. Additionally, the insulation layer shields of the integral electrodes and signals from external environment, ensuring system stability and reliability under practical conditions. The high resistance fabric used in the insulation layer is made from polyester, a synthetic polymer (polyethylene terephthalate) that is inexpensive, widely available. In this fabric, the polyester fibers are tightly knitted, providing structural stability as well as a smooth, soft, and cool surface texture. The material adopts a controlled two-way knit structure, allowing horizontal stretch while maintaining minimal vertical stretch. This design offers flexibility in one direction while ensuring mechanical and dimensional stability in the other, which is essential for maintaining consistent electrode positioning and fabric integrity during use. This design provides flexibility in one direction while preserving mechanical and dimensional stability in the other, ensuring consistent electrode positioning and preventing deformation during use. Owing to its inherently high electrical resistance, combined with durability, chemical resistance, and stable mechanical properties, the polyester fabric offers a reliable insulating layer that supports effective and unobtrusive capacitive ECG sensing within the mattress system.

We also integrate a thin sponge support structure into the electrodes design. Due to the flexibility and cushioning effect, this deformable supporting material serves to alleviate pressure variations caused by slight movements or muscle contractions. Moreover, it aids in conforming the electrodes more closely to the contours of the human body, thereby reducing contact impedance and enhancing signal quality [36]. All the layers of the mattress are bonded through thermal lamination using hot-melt adhesive to ensure uniform adhesion across the surface. The edge regions are additionally reinforced with mechanical quilting and stitching to enhance structural stability and prevent separation during use. This multiple layer configuration ensures that the capacitive coupled active electrodes are both sensitive and comfortable for long term use in monitoring bioelectric signals.

The integration of electrodes plays a critical role in enhancing both the performance and comfort of the cECG mattress system. Because the electrodes are mainly made from textile which naturally adapt to the contours of the body, ensuring more consistent capacitive coupling across different sleeping postures. This flexibility helps maintain stable electrode–body spacing, reducing signal fluctuations caused by micro-movements. Additionally, the breathable and smooth fabric surface significantly improves user comfort, minimizing tactile awareness and eliminating the skin irritation commonly associated with adhesive electrodes. The high-resistance textile structure further supports reliable signal acquisition without compromising the softness of the bedding interface. Together, these characteristics make the whole fabric electrodes suited for unobtrusive, long-term cardiac monitoring in real-world sleep environments.

### 2.3. Signal Acquisition and Transmission Unit

The overall circuit design is divided into two main sections based on functionality: signal acquisition and data transmission.

**Figure 4:**
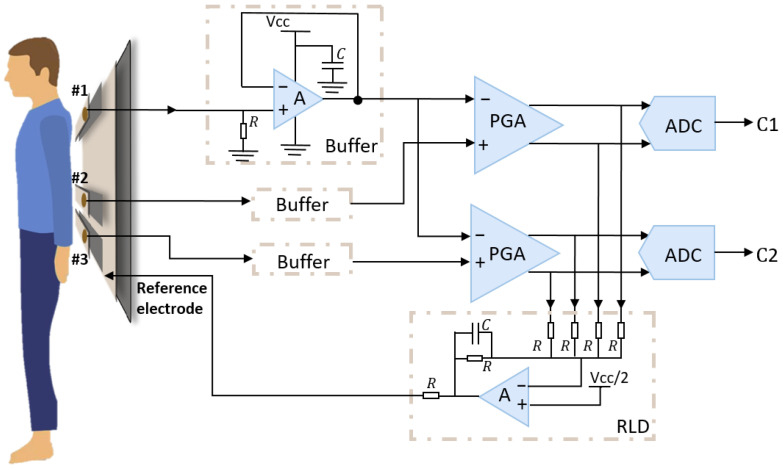
Schematic of signal acquisition with mattress electrodes.

In the signal acquisition section, the main focus is on acquiring and processing physiological signals. This unit involves components such as differential amplifiers, analog-to-digital converters (ADCs), and right leg drive (RLD) circuitry. Capacitive coupled active electrodes, positioned under the clothing, sense electrical signals of skin’s surface. These signals are then processed through the buffer circuitry of the signal acquisition module, enhancing the immunity to interference and load capacity. Voltage follower is used as the buffer to increase input impedance, as shown in Figure 4. Subsequently, the signals are routed to a programmable gain amplifier (PGA) in the analog front-end for further amplification and differential processing of signals from two electrodes, resulting in the generation of electrocardiogram signals. The right leg drive (RLD) circuitry is a conventional but rather effective technique used to minimize common-mode interference, which can occur due to power line noise and other environmental sources [37]. The common-mode voltage is tracked and fed back to the body through the reference electrode, effectively constraining the common-mode movement to a narrow range. In our setup, the reference electrode is positioned beneath the three active electrodes and is electrically insulated from them to minimize direct coupling. While indirect contact with the body may occur through clothing, this arrangement was chosen to balance user comfort, mattress geometry, cable routing, and common-mode noise suppression, while keeping the multilayer mattress structure mechanically simple. Regarding the concern about oscillations, our tests showed no observable instability under normal operating conditions, indicating that the RLD feedback loop remained stable. The RLD injection and active shield are implemented through this shared reference electrode to minimize wiring complexity.

Finally, ADCs convert the analog ECG signals into digital form. The functionalities of differential amplification, analog-to-digital conversion, and right leg drive are implemented using integrated circuit ADS1292 (Texas Instruments Inc., Dallas, TX, USA). The ADS1292 is a two-channel, simultaneous-sampling, 24-bit delta-sigma ADC with a built-in PGA. Its integration facilitates the development of a system characterized by exceptional performance and high level of integration.

The data transmission unit manages the transfer of processed signals to external devices for analysis and interpretation. This unit includes a microcontroller (MCU) and communication modules. The MCU oversees data transmission, handling tasks such as data formatting, storage, and communication protocol management. The communication modules enable wireless transmission of the data to external devices, facilitating real-time monitoring or remote analysis. Once the signal acquisition process is complete, the data is transmitted to the data transmission section. The ECG digital data is conveyed to the STM32 microcontroller unit through a serial peripheral interface (SPI) port. To enhance processing efficiency, the STM32 employs direct memory access (DMA) to store the SPI data for the two cECG channels. Upon accumulating a packet of data, the STM32 arranges it using a custom communication protocol. Subsequently, the data is transmitted via serial communication to the ESP12F WiFi module (Espressif Systems Company Ltd., Shanghai, China). The ESP12F module is designed based on the ESP8266 chip and includes essential external components. Through this WiFi module, the data is transferred to the data display and storage unit using the TCP/IP protocol. In this scenario, the signal acquisition and transmission unit is configured as the client, while the data display and storage unit act as the TCP server. By establishing this setup, the acquired cECG data undergoes efficient processing and transmission, enabling seamless communication between the acquisition unit and the data display and storage unit over a wireless network.

### 2.4. Signal Processing Methods

Conventional ECG denoising methods includes Wavelet Transform (WT), Empirical Mode Decomposition (EMD), adaptive filtering and so on. We apply a novel denoising approach for cECG signals based on Adaptive Fourier Decomposition (AFD), enhancing denoising efficiency and signal quality. The AFD algorithm integrates traditional Fourier decomposition with a greedy strategy [38]. By dynamically generating adaptive orthogonal basis functions that align with the signal characteristics, the target signal is decomposed into a series of mono-components (MCs) that only contain non-negative analytic phase derivatives [39]. Compared to conventional decomposition methods that rely on pre-defined basis functions, AFD achieves rapid energy convergence through signal-driven optimal basis selection [40]. This enables effective separation of the pure signal and noise when they share overlapping frequency ranges but exhibit different energy distributions [41]. Additionally, the AFD algorithm is grounded in a rigorous mathematical framework, with its convergence strictly proven within the Hardy space [38]. Owing to its efficient decomposition and reconstruction capabilities, AFD and its variants have been successfully applied across various domains [39,40]. Previous studies have demonstrated that in denoising tasks involving both real ECG signals from the MIT-BIH Arrhythmia Database and synthetic ECG signals, AFD outperforms traditional methods in terms of signal to noise ratio improvement and waveform fidelity [41].

The AFD-based denoising process for cECG signals proposed in this study includes the following key steps. Suppose that the noisy cECG signal is expressed as s(t), as shown in Equation (1).

(1) s(t)=h(t)+w(t)

where h(t) denotes the pure signal to be recovered, and w(t) represents independent noise with energy lower than that of h(t).

Then the noisy cECG signal s(t) undergoes a Hilbert transform, projecting it to the $H^{2}$ space to obtain the analytic representation G(t). According to the AFD algorithm, G(t) is decomposed and expressed as shown in Equation (2).

(2) $$G\left(t\right)=\sum_{n=1}^{N}G_{n}\left(e^{jt}\right)B_{n}\left(e^{jt}\right)+G_{N}\left(e^{jt}\right)$$

where $G_{n}$ represents the reduced remainders, and $B_{n}$ denotes the basis functions.

(3) $$G_{n}\left(e^{jt}\right)=R_{n-1}\left(e^{jt}\right)\prod_{l=1}^{n-1}\frac{1-{\bar{a}}_{l}e^{jt}}{e^{jt}-a_{l}}$$

(4) $$B_{n}\left(e^{jt}\right)=\frac{\sqrt{1-{\left|a_{n}\right|}^{2}}}{1-{\bar{a}}_{n}e^{jt}}\prod_{k=1}^{n-1}\frac{e^{jt}-a_{k}}{1-{\bar{a}}_{k}e^{jt}}$$

By combining Equations (2) and (3), G(t) can be expressed in terms of the reduced remainders $G_{n}$ and is reformulated as shown in Equation (5).

(5) $$G\left(t\right)=\sum_{n=1}^{N}\langle G_{n},e_{\left\{a_{n}\right\}}\rangle B_{n}\left(e^{jt}\right)+G_{N+1}\left(e^{jt}\right)\prod_{n=1}^{N}\frac{e^{jt}-a_{n}}{1-{\bar{a}}_{n}e^{jt}}$$

The stop criterion of the AFD is given in Equation (6).

(6) $$\left.\min_{N>1,N\in Z}\left\{\left|\;\frac{{∥s\left(t\right)∥}^{2}}{∥\hat{h}{\left(t\right)∥}^{2}}-\left(1+\frac{1}{10^{S_{SNR}/10}}\right)\right.\right|\right\}$$

When this value reaches the minimum, the computation stops, and the reconstructed filtered cECG signal is obtained, as shown in Equation (7).

(7) h^←Re∑n=1NGn,eanBn

**Figure 5:**
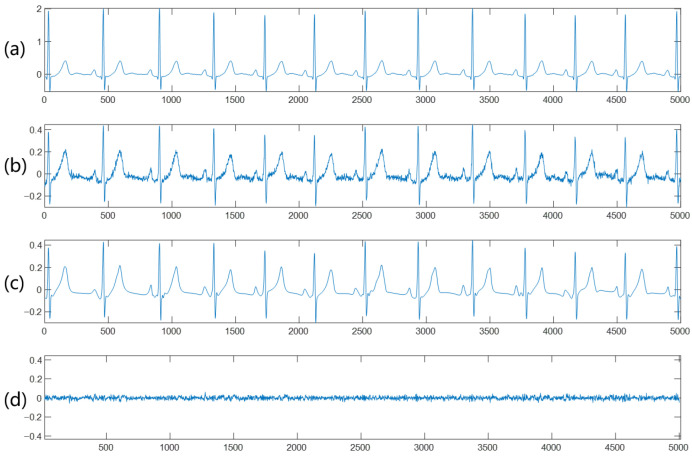
cECG denoising performance of the AFD-based denoising method. (**a**) Gold standard Holter ECG signal. (**b**) Original cECG signal. (**c**) Denoised cECG signal based on proposed method. (**d**) Absolute error of the original cECG and denoised signal.

Figure 5 shows the cECG denoising performance of the AFD-based denoising method.

## 3. Experiment and Results

### 3.1. Experimental Setup

**Figure 6:**
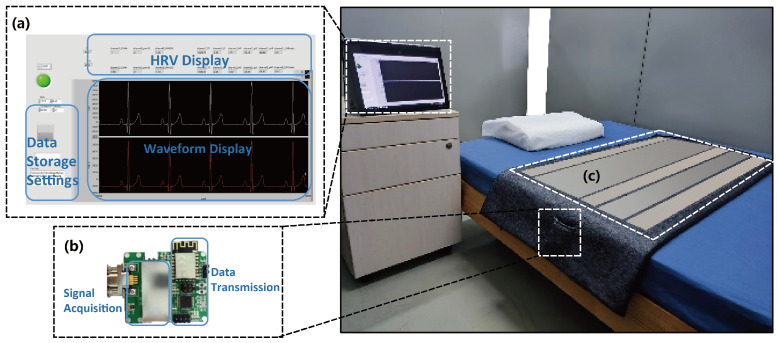
System prototype. (**a**) Waveform display interface. (**b**) Signal acquisition and data transmission circuit. (**c**) Mattress electrode.

To quantitatively assess the monitoring performance of the system, the proposed capacitive ECG mattress system and filtering algorithms were evaluated with simultaneously clinical single lead ECG measurements. The mattress system used in the experiment is shown in Figure 6. Fifteen healthy volunteers were recruited for the experiment, including 10 male participants and 5 female participants. The average age is 27.7 years (SD = 3.5 years), the average weight is 63.8 kg (SD = 9.1 kg), the average height is 170.5 cm (SD = 7.8 cm), and the average BMI is 21.89 (SD = 2.28). The detailed information of the participants is shown in Table 1. All participants provided written informed consent prior to their involvement in the study. The consent process ensured that participants were thoroughly informed about the study’s purpose, procedures, risks, and benefits. The test was approved by the ethics committee of Fudan University (approval number: FE23281I).

**Figure 7:**
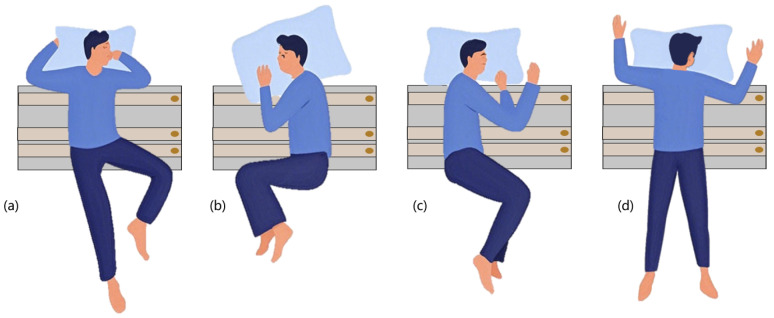
Sleep postures (**a**) Supine. (**b**) Right lateral. (**c**) Left lateral. (**d**) Prone.

In the experiment, participants were instructed to lie on a bed equipped with the proposed mattress system while wearing a gold-standard Holter monitor for approximately 20 min in a resting state. The participants were asked to alternate four sleeping postures include supine, right lateral, left lateral, and prone, adopting their naturally comfortable sleeping postures. An example of the four postures with the mattress is shown in Figure 7. Participants were instructed to maintain each posture as they naturally would during actual sleep, remaining still without movement for a minimum of 3 min. In this study, each participant was instructed to adopt their most comfortable sleeping posture to better approximate realistic sleep conditions. Acknowledging that sudden body movements, such as turning over or repositioning, can generate motion artifacts several orders of magnitude larger than the cECG signal, we carefully monitored participant movements throughout the experiment. These events were documented in real time, and the corresponding contaminated signal segments were excluded manually. This approach ensured that only physiologically meaningful and posture-stable cECG data were analyzed. Although natural sleep involves multiple posture changes, individuals spend most of the time in relatively stable positions. This study focuses specifically on evaluating signal acquisition quality during these stable phases across different sleeping postures.

The FDA (Food and Drug Administration, Silver Spring, MD, USA) and NMPA (National Medical Products Administration, Beijing, China) approved Holter recorder BI9900 (Biomedical Instruments, Shenzhen, Guangdong, China) was used. It was simultaneously used as a reference for standard cardiac examinations. Both systems operated at a sampling rate of 500 Hz. Participants were instructed to wear cotton shirts throughout the experiment. When the experiment finished, each participant was asked to evaluate wearability, ease of use, and long-term user compliance using a continuous Visual Analog Scale (VAS). The laboratory technician recorded the corresponding values of the VAS.

### 3.2. Evaluation Methods

We systematically evaluated the proposed cECG mattress system by quantifying the waveform characteristics of physiological structures in cECG and comparing them with the corresponding waveforms from the clinical gold-standard Holter monitor.

Firstly, the processed recordings were divided into non-overlapping 10 s segments to facilitate evaluation and comparison. Then the noise segments caused by body movements, such as turning over, were discarded manually. In this study, 16,817 heartbeats in 1751 segments in total were used to evaluate the proposed mattress system.

Next, a quantitative evaluation and comparison of the system were conducted. The measurement evaluation is divided into two components: the positions of key waveform features and the durations of critical intervals. The key waveform features analyzed include the P wave, QRS complex wave, and T wave, while the important intervals include the RR interval, PR interval, and RT interval. The RR interval is defined as the time between the peaks of consecutive R waves. The PR interval is measured within a single cardiac cycle from the peak of the P wave to the peak of the R wave. Similarly, the RT interval is measured within a single cardiac cycle from the peak of the R wave to the peak of the T wave. We utilized an open-source MATLAB (R2023a) toolbox ECGdeli [42] to automatically detect the positions of the waves of interest in both cECG and Holter ECG recordings. The duration of RR, PR and RT interval was subsequently calculated by subtracting the positions of the corresponding waveform peaks. A wave was considered a true positive detection if it was identified in both cECG and Holter ECG within a time tolerance of ±75 ms, according to Association for the Advancement of Medical Instrumentation recommended practice for testing and reporting performance results of ventricular arrhythmia detection algorithms (AAMI-ECAR) guidelines [43,44,45]. To comprehensively assess the differences between cECG system and Holter, this study evaluates waveform detection and interval measurements using true positive rate (TPR), mean absolute error (MAE), and root mean square error (RMSE). Additionally, boxplots are employed to visualize deviations in waveform positions, while Bland-Altman plots provide a graphical representation of interval measurement distributions, facilitating an assessment of agreement between the two systems.

### 3.3. System Performance

**Figure 8:**
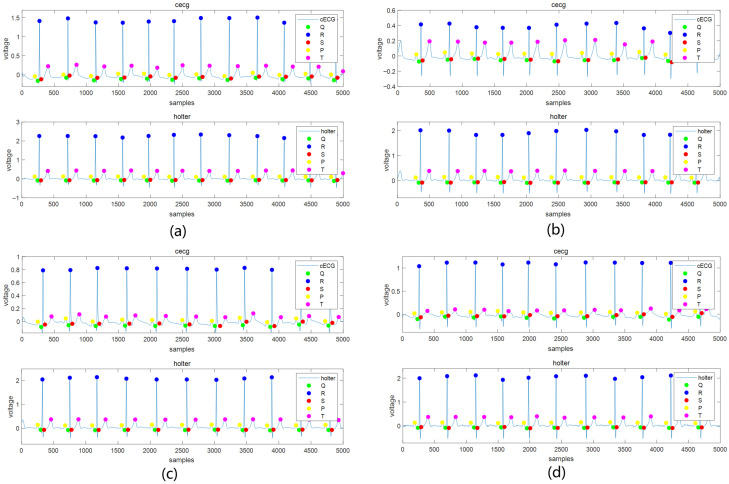
Comparison of cECG signal and holter signal waveforms in four sleeping positions. (**a**) Supine. (**b**) Right lateral. (**c**) Left lateral. (**d**) Prone.

**Figure 9:**
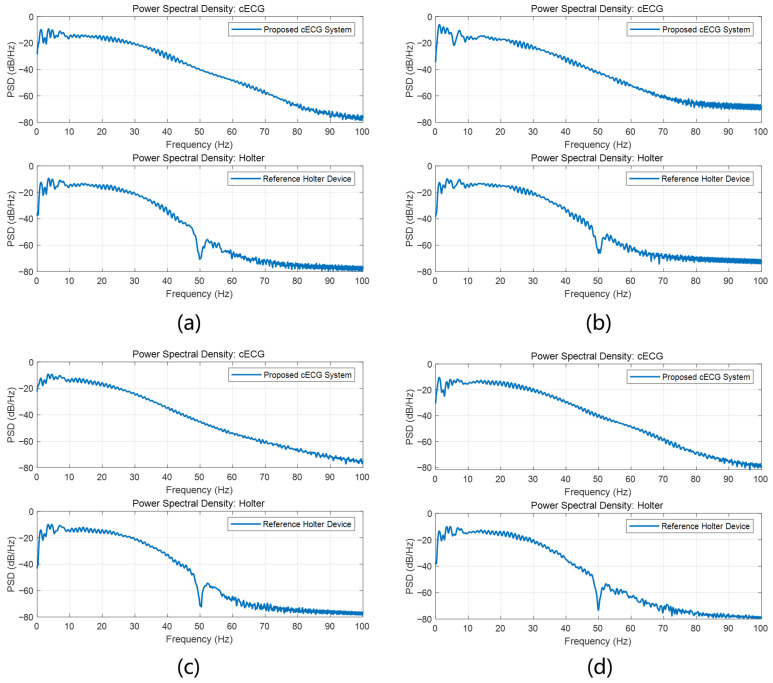
Power spectral density comparison of cECG signal and holter signal waveforms in four sleeping positions. (**a**) Supine. (**b**) Right lateral. (**c**) Left lateral. (**d**) Prone.

After AFD processing, the collected signals were analyzed using the open-source waveform extraction algorithm ECGdeli to identify the time locations of P, R, and T waves in both cECG and Holter ECG. The MAE and RMSE were then computed for these waveform locations. Subsequently, the RR, PR, and RT intervals were determined, and their respective RMSE and MAE values were calculated to evaluate the accuracy of interval measurements. For each participant, after removing noisy data, an average of 116 10-s segments were retained, encompassing a total of 16,817 heartbeats. The detailed information on the number of segments and heartbeats for each subject is provided in Table 2. Figure 8 presents a morphological comparison of cECG and Holter ECG waveforms across four representative signal segments, corresponding to the supine, right lateral, left lateral, and prone sleeping positions. Frequency-domain analysis are performed on both cECG signal and reference Holter ECG signals across four sleep postures. Power spectral density (PSD) was estimated using the Welch method. The PSD estimation results of each posture, as shown in Figure 9, demonstrate that the system maintains signal integrity across the relevant physiological frequency range, confirming its effective bandwidth and consistency with expected characteristics.

Table 3 presents the average of evaluation results for P wave, R wave, T wave, PR interval (PRI), RR interval (RRI), and RT interval (RTI) across different body postures. The table includes key performance metrics, namely MAE, RMSE, and TPR, providing a comprehensive assessment of the system’s accuracy and reliability in waveform detection and interval measurement.

The TPR for R waves across all postures is 1, and the TPR for P and T waves is nearly 1, indicating that changes in posture have minimal impact on wave recognition. The R wave exhibits a MAE of 1.12 ms and a RMSE of 1.71 ms, while the P wave shows an MAE of 7.89 ms and an RMSE of 12.17 ms, and the T wave has an MAE of 7.88 ms and an RMSE of 12.81 ms. These results indicate that both systems successfully identify the presence of each wave, with minimal impact from posture variations.

The results for the intervals show that both systems exhibit relatively small errors in calculating the intervals. For the RRI, the MAE is 0.89 ms, with an RMSE of 1.46 ms; for the PRI, the MAE is 7.77 ms, with an RMSE of 12.11 ms; and for the RTI, the MAE is 7.85 ms, with an RMSE of 12.75 ms. These findings suggest that the errors in interval calculation are minimal, further demonstrating the accuracy and reliability of the systems in measuring key heart rate parameters. The small discrepancies indicate that the cECG mattress system performs well, even in tasks such as interval computation, which are crucial for accurate cardiac assessment.

**Figure 10:**
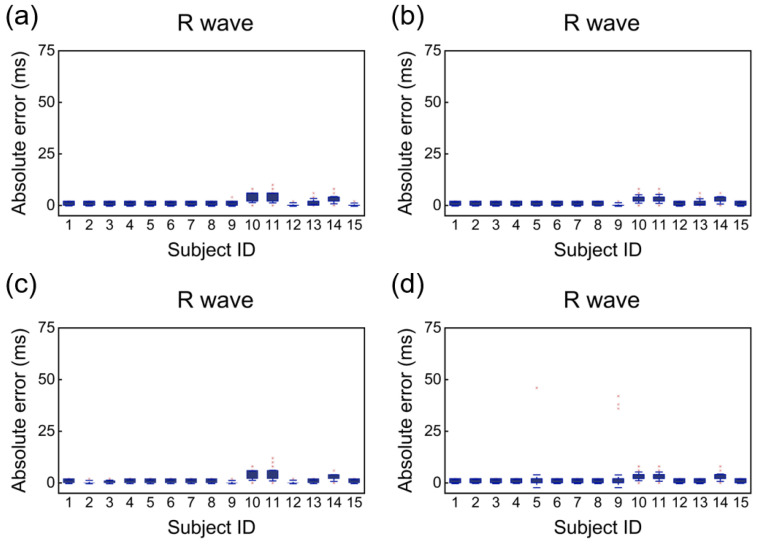
Absolute error of R waveforms in four sleeping positions. (**a**) Supine. (**b**) Right lateral. (**c**) Left lateral. (**d**) Prone.

Figure 10 presents the absolute error of R wave detected simultaneously by the cECG mattress and the standard Holter monitor across four different sleep positions: supine, right lateral, left lateral, and prone. The comparative analysis reveals that the R wave measured by cECG mattress exhibits minimal error relative to the clinical Holter monitor, with the mean error approaching zero. This indicates that R wave detection using cECG mattress closely aligns with the results obtained from the Holter monitor.

**Figure 11:**
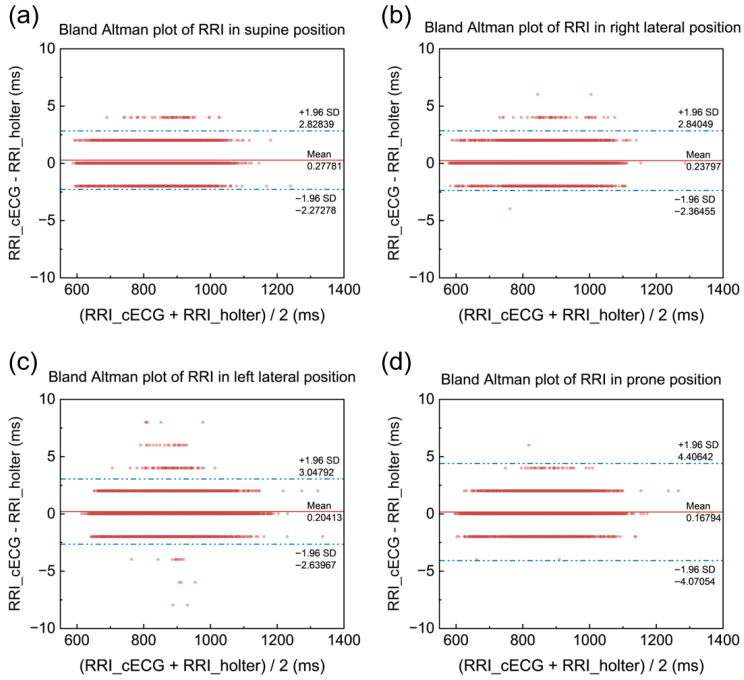
Bland–Altman plots of RRI. (**a**) Supine. (**b**) Right lateral. (**c**) Left lateral. (**d**) Prone.

The Bland–Altman analysis of RR intervals obtained from cECG mattress and the Holter across four positions is shown in Figure 11. The plot shows that the vast majority of data points fall within the ±1.96 standard deviation range, indicating a high level of agreement between cECG and Holter measurements of RR intervals. This result further confirms that cECG system can reliably capture RR interval data comparable to Holter, making it suitable for ECG monitoring.

**Figure 12:**
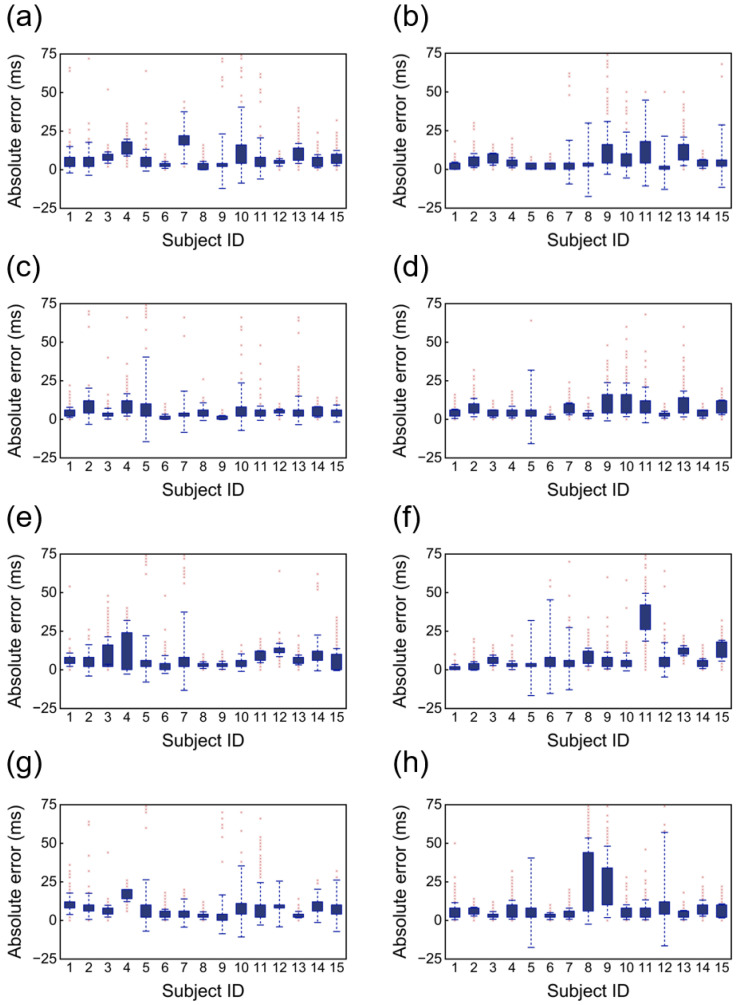
Absolute error of P and T waveforms in four sleeping positions. (**a**,**c**,**e**,**g**) is P wave, (**b**,**d**,**f**,**h**) is T wave in supine, right lateral, left lateral, and prone positions, respectively.

Figure 12 presents box plots of the absolute errors in P wave and T wave detection by cECG system and Holter across four sleeping positions. Compared to R wave detection, the error in P wave and T wave detection using cECG is slightly larger. However, the majority of errors remain within 75 milliseconds, which falls within the widely accepted clinical standard. These findings suggest that P-wave and T-wave detection by cECG remains within an acceptable range, demonstrating its high accuracy in measuring these waveforms.

**Figure 13:**
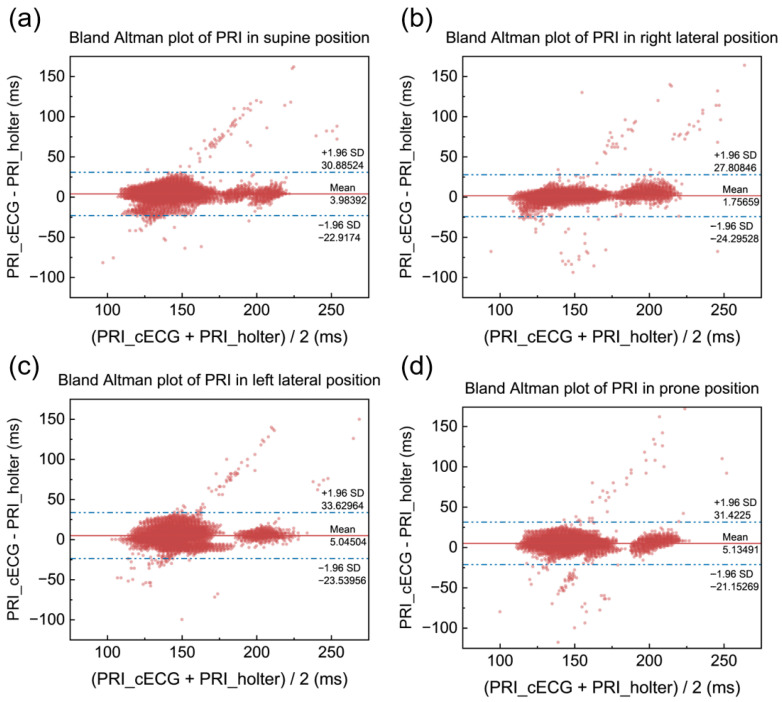
Bland–Altman plots of PRI. (**a**) Supine. (**b**) Right lateral. (**c**) Left lateral. (**d**) Prone.

**Figure 14:**
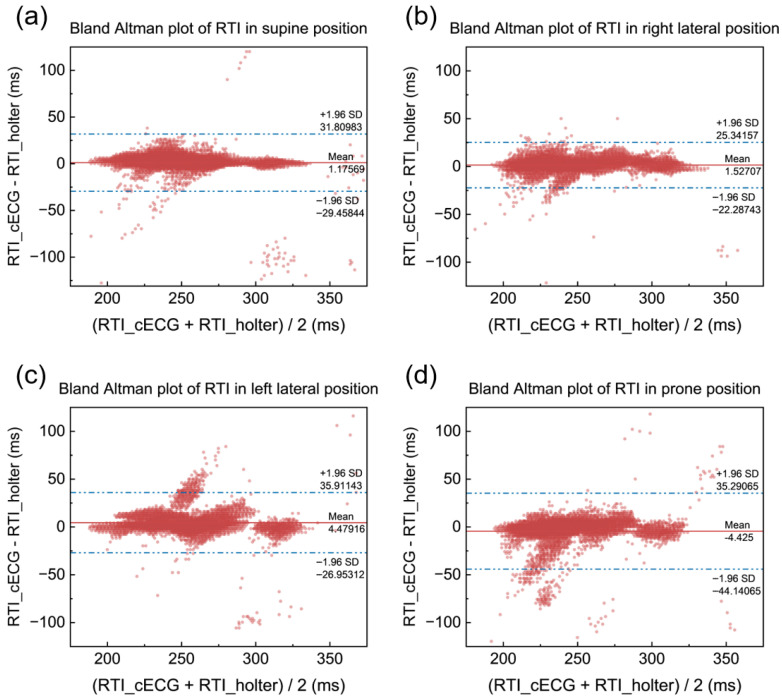
Bland–Altman plots of RTI. (**a**) Supine. (**b**) Right lateral. (**c**) Left lateral. (**d**) Prone.

The Bland–Altman plots for PR interval and RT interval measurements by cECG and the Holter device are shown in Figure 13 and Figure 14, respectively. These plots demonstrate a high level of agreement between cECG and Holter measurements for both intervals. Additionally, the PR interval values are mostly distributed between 100 ms and 300 ms, while the RT interval values are concentrated between 200 ms and 400 ms. These results align with actual physiological data, further confirming the accuracy and reliability of cECG systems in measuring the bioelectrical signal’s intervals.

### 3.4. User Experience Evaluation

**Figure 15:**
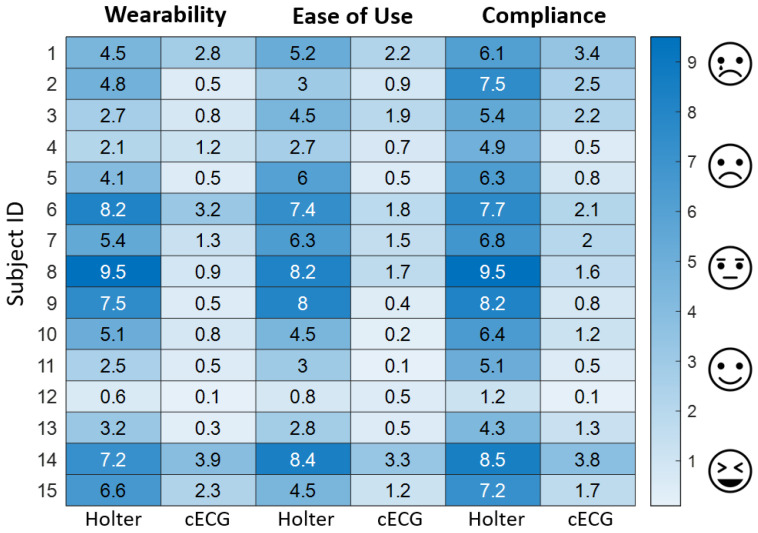
Visual Analog Scale (VAS) degree of two device.

The subjective user experience of both the cECG mattress system and the Holter device was evaluated using a continuous Visual Analog Scale across three key dimensions: wearability, ease of use, and long-term user compliance. The evaluation aimed to capture participants’ perceptions of comfort, usability, and acceptance, providing a comprehensive comparison between the two systems. The result is shown in Figure 15.

(1) Wearability Assessment: Participants rated the wearability of each system based on comfort, physical fit, and potential skin reactions. The unobtrusive nature of the mattress system allowed for passive monitoring without direct skin contact, whereas the Holter device required electrode attachment, which could cause discomfort and irritation.

(2) Ease of Use Evaluation: VAS were also utilized to assess the ease of system setup, operation, and adjustments. The mattress system required minimal user interaction, as it functioned autonomously once placed on the bed. In contrast, the Holter device necessitated manual placement of electrodes and attachment of wires, which could be perceived as cumbersome, especially for long-term monitoring.

(3) Long-Term Compliance and User Acceptance: To evaluate user compliance and acceptance, participants were asked to rate their willingness to continue using each system over an extended period. Given its non-intrusive design and seamless integration into daily routines, the mattress system received significantly higher acceptance scores compared to the Holter device, which users found restrictive and inconvenient for prolonged use.

We used the Wilcoxon signed-rank test to analyze the differences across the three dimensions between the Holter and cECG mattress, with p value correction applied using the Bonferroni method. The results demonstrated significant differences between Holter and cECG mattress in terms of wearability (mean difference = 3.5, *p* = 7.2 × 10^−4^), ease of use (mean difference = 3.9, *p* = 6.1 × 10^−5^), and long-term compliance (mean difference = 4.8, *p* = 7.2 × 10^−4^). Holter received significantly higher scores than cECG mattress (*p* < 0.001).

The capacitive mattress system consistently outperformed the Holter device across all three subjective dimensions: wearability, ease of use, and long-term compliance. Participants reported a higher level of comfort and usability, making the mattress-based system a promising alternative to conventional commercial devices, particularly for applications requiring continuous, long-term physiological monitoring.

### 3.5. Durability Test

**Figure 16:**
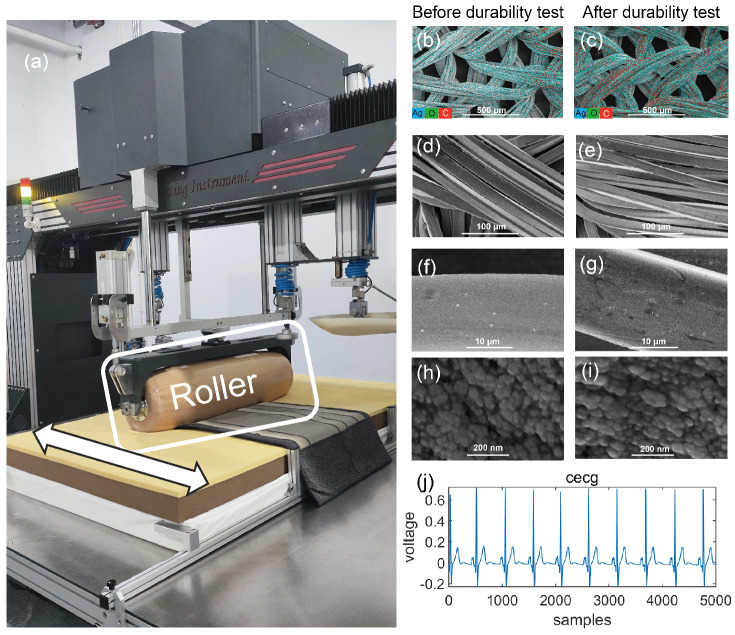
Durability test. (**a**) Roller equipment. (**b**,**c**) EDS images of the fabric electrodes before and after durability testing, respectively. (**d**–**i**) are SEM images: (**b**,**d**,**f**,**h**) represent fabric electrodes samples before testing; (**c**,**e**,**g**,**i**) represent samples after durability testing. (**j**) shows the cECG signal recorded after the durability test.

To ensure the long-term monitoring performance of the capacitive ECG mattress, a durability test was conducted in accordance with the European Standard BS EN 1957:2012—Furniture—Beds and mattresses—Test methods [46] for the determination of functional characteristics and assessment criteria. The test was performed using specialized roller equipment, which includes a cylindrical roller and a mechanism designed to move it horizontally across the mattress surface. The equipment of the durability test is shown in Figure 16a. The setup simulates prolonged usage conditions. Under static conditions, the roller applied a load of 1400 ± 7 N to the mattress, with a rotational moment of inertia of 0.5 ± 0.05 kg·m^2^. The roller conform to the mattress surface and could move freely in the vertical direction to adapt to surface. The roller movement followed a symmetrical, approximately sinusoidal trajectory along the longitudinal centerline of the mattress. The durability test consisted of 30,000 cycles, with the roller operating at a frequency of 16 ± 2 cycles per minute, simulating real-world usage conditions.

After completing the durability experiment, further analyses were conducted. Energy-dispersive X-ray spectroscopy (EDS) layered image revealed that unused and post-test fabric electrodes contained the same three characteristic elements: silver, oxygen, and carbon. The EDS image of the sample before the durability test is shown in Figure 16b, while the EDS image of the sample after the durability test is presented in Figure 16c. Additionally, scanning electron microscopy (SEM) was employed to perform a comparative analysis of the flexible fabric electrodes, enabling a detailed examination of the morphological differences between unused electrodes and those subjected to the durability test. Figure 16d,f,h show the electrode samples before durability test, while Figure 16e,g,i display the samples after the durability test. The microscopic surface morphology was observed and compared at three different scales: 100 μm, 10 μm, and 200 nm. The cECG signal recorded after the durability test, shown in Figure 16j, remained comparable to those obtained before the test. These findings indicate that the physical structure, chemical composition and the distribution pattern of the flexible fabric electrodes remained stable during long-term use, thereby preserving reliable electrophysiological monitoring performance.

## 4. Discussion

The capacitive ECG measurement system holds significant potential for dynamic and unobtrusive cardiac health assessment. Unlike traditional clinical wet electrodes, which rely on direct skin contact and conductive gel to ensure stable signal acquisition, capacitive fabric electrodes operate without direct skin contact. While capacitive fabric electrodes offer advantages in terms of long-term comfort, ease of use, and potential for unobtrusive integration, it is more susceptible to various noise sources compared to traditional wet electrodes [5,6,10]. These may degrade signal quality and pose challenges for accurate morphological analysis and clinical applications. In this study, we proposed a novel capacitive ECG mattress system and conducted experimental evaluations to assess its feasibility for morphological analysis and clinical parameter extraction. We designed a multi-layered capacitive ECG mattress system integrated with an AFD filtering method to effectively suppress noise while preserving essential cECG features. Through efforts in fabric electrode integration, hardware design, and algorithm implementation, the system enhances the clarity of P, QRS, and T waveforms, which are critical for clinical evaluation. The proposed multi-layered mattress system offers a non-invasive platform for continuous cardiac monitoring. Unlike traditional Holter monitors or adhesive electrodes, this system can be integrated into everyday sleeping or resting environments, enhancing user comfort and compliance for long-term monitoring. Such continuous, passive monitoring has the potential to facilitate early detection of arrhythmias and other cardiac anomalies that might be missed in episodic clinical assessments. Moreover, its deployment in hospitals, long-term care facilities, or home settings may provide clinicians with richer, longitudinal datasets, supporting more informed decision-making and personalized patient care.

Our experimental results demonstrate a high level of consistency between capacitive ECG and clinical Holter ECG, underscoring the potential of capacitive electrodes for cardiac monitoring in capturing key morphological features. Moreover, the user experience evaluation demonstrated that the system offers significant advantages in user comfort, ease of use, and long-term compliance. Additionally, the durability testing confirmed that the mattress system maintains reliable performance over extended periods of use. This is the key contribution of our research. By capturing high-quality cECG signals through the flexible fabric electrodes without direct skin contact, this mattress system provides a solution for long-term, unobtrusive, continuous, and comfortable cardiac monitoring in everyday environments. In most cases, the cECG waveform detection rate TPR is close to 1, demonstrating stable and reliable performance. R wave detection is highly accurate, with a TPR of 1 and an MAE of 1.12 ms. P and T wave measurements exhibit slightly lower precision, they still achieve a high TPR of 0.99. Among different body positions, the right lateral posture yielded the smallest errors, with P wave, T wave, PRI, and RTI measurements performing optimally. The minimal differences across other positions suggest that body posture has little impact on detection accuracy in the proposed system.

The experimental results indicate that the cECG mattress system achieves high accuracy in R wave detection, while P and T wave identification is slightly less accurate. The higher amplitude and steeper slope of the R wave provide stronger resistance to noise, making it easier to detect. In contrast, the lower amplitude of P and T waves makes them more susceptible to noise. When using cECG mattress for testing, the presence of clothing further obscures P and T waves, making them more difficult to capture [45]. When using a cECG mattress, the presence of clothing between the body and the electrode further attenuates these low-amplitude component. Despite these challenges, instances where P and T waves were distinguishable support the system’s potential for tracking higher-level cardiac dynamics. Generally, R wave detection algorithms are well-established and exhibit higher accuracy, whereas P wave and T wave detection is more challenging due to their lower amplitudes and susceptibility to noise. In this study, we utilized basic open source tools for preliminary waveform recognition and localization. Advanced techniques may be required to improve P and T waves detection. Future research could focus on developing specialized waveform recognition or information recover algorithms [47] optimized for the cECG signals, enhancing detection accuracy and reliability. These insights reinforce the need for continued refinement of both hardware and signal processing strategies to enhance the reliability of full-waveform analysis in future iterations of the system.

In our prototype, the RLD electrode is placed beneath the sensing region. Alternative arrangements including place the RLD electrode farther from the sensing area tends to reduce parasitic capacitance seen by the active front end and thereby help preserve the high input impedance and bandwidth of the impedance followers, although it may necessitate longer wiring. Another alternative approach is to split the reference into multiple driven shields surrounding each sensing zone; such segmented driven shields can locally neutralize electrode-to-environment capacitances and reduce contamination of the differential signal. Future work will systematically compare these advanced configurations [48,49] using objective measures such as CMRR, input capacitance, noise spectral density, and oscillation margin, alongside practical factors (comfort and wiring complexity), to determine the optimal configuration for mattress-based cECG.

In future research, collecting whole night sleep data can provide more comprehensive insights. Since the typical application of the mattress system is during sleep, unavoidable body movements such as turning over will cause significant waveform variations. Effectively recognizing and filtering out these interference signals requires the development of a highly efficient cECG quality classification algorithm. Such algorithm would not only significantly improve data processing efficiency but also enable a more accurate assessment of the system’s performance in practical applications. Therefore, whole night sleep tests and advancements in automatic quality classification algorithms are crucial directions for future research. Several efficient quality classification methods for ECG signals already exist [50]. These methods can automatically identify high-quality and low-quality signals, reducing manual intervention and improving processing efficiency. Integrating these advanced algorithms into the mattress cECG system could significantly enhance its adaptability in real-world environments, particularly in handling significant signal interference during sleep [51].

At present, the proposed mattress system has been tested only on healthy volunteers. Clinical validation involving patients with pathological conditions is part of planned future work. Nevertheless, the current results indicate that the system maintains sufficient performance to support studies of pathological signals. Future studies should focus on designing experiments with patients with cardiovascular conditions to evaluate the system’s performance and clinical applicability under complex pathological scenarios. Applying the system in clinical diagnostics and verifying its use in real medical environments will provide a clearer assessment of its potential, particularly for early diagnosis. Additionally, integrating low-power edge-AI for on-device ECG analysis [52] and leveraging IoT-based remote monitoring in daily-life settings [53] could further enhance the practicality and applicability of the unobtrusive monitoring system. However, these works are not the focus of this study, yet they offer valuable directions for future research.

## 5. Conclusions

Our study introduces a capacitive ECG mattress system designed to improve comfort and accuracy in long-term monitoring scenarios. By leveraging a multi-layered structure comprising flexible fabric active electrodes, signal acquisition circuits, and dedicated signal processing algorithms, our system demonstrates the ability to accurately monitor cECG waveforms across various sleeping positions. Importantly, the proposed cECG mattress system, comparable to medical-grade devices, exhibits high accuracy and reliability in detecting and analyzing cardiac waveforms. With the true positive rate of 0.99, the system ensures robust waveform detection cross various body positions. The low mean absolute error values (1.12 ms for the R wave, 7.89 ms for the P wave, and 7.88 ms for the T wave) demonstrate accurate waveform extraction. Furthermore, the system maintains a low MAE of 0.89 ms for the RR interval, 7.77 ms for the PR interval, and 7.85 ms for the RT interval, confirming its reliability in interval measurements. These results indicate that the signal obtained from the cECG mattress system is sufficient for accurately identifying critical waveform features and interval durations. These findings underscore the potential of our cECG mattress system to bridge the gap between research and clinical applications in cECG monitoring. By prioritizing user comfort and signal accuracy, our system offers a promising solution for enhancing the efficacy and feasibility of long-term monitoring efforts.

## Figures and Tables

**Table 1:** Subject Information.

Subject ID	Age	Gender	Height (cm)	Weight (kg)	BMI
1	28	Male	172	82	27.71
2	31	Male	185	70	20.45
3	24	Male	175	73	23.83
4	25	Male	174	74	24.44
5	31	Female	163	50	18.81
6	24	Male	180	75	23.14
7	29	Female	160	56	21.87
8	33	Female	161	52	20.06
9	27	Male	178	67	21.14
10	24	Male	168	54	19.13
11	33	Male	166	60	21.77
12	24	Female	161	57	21.98
13	26	Male	168	65	23.03
14	24	Male	182	63	19.01
15	33	Female	165	60	22.03

**Table 2:** Number of Segments and Heartbeats in Different Positions.

Subject ID	Number of Segments				Number of Heartbeats			
	Supine	Right	Left	Prone	Supine	Right	Left	Prone
1	33	30	33	29	300	311	316	269
2	32	30	32	29	299	295	270	247
3	29	28	29	29	299	283	272	271
4	37	24	27	32	478	314	335	389
5	27	53	24	27	221	452	186	233
6	31	28	20	22	287	231	155	204
7	24	39	36	34	256	373	361	352
8	26	27	33	30	257	259	314	289
9	31	27	35	53	290	253	402	507
10	27	20	22	24	243	183	198	224
11	25	26	31	21	242	259	289	203
12	27	18	37	30	217	142	272	244
13	28	36	35	32	306	379	390	365
14	21	19	18	19	171	154	148	160
15	37	29	29	30	363	286	266	283

**Table 3:** Performance metrics for different positions.

Position	P Wave			R Wave			T Wave			PRI		RRI		RTI	
	MAE (ms)	RMSE (ms)	TPR	MAE (ms)	RMSE (ms)	TPR	MAE (ms)	RMSE (ms)	TPR	MAE (ms)	RMSE (ms)	MAE (ms)	RMSE (ms)	MAE (ms)	RMSE (ms)
Supine	8.38	12.52	0.99	1.18	1.71	1	6.86	13.31	0.99	8.13	12.34	0.86	1.33	6.78	13.23
Right lateral	5.54	9.69	0.99	1.15	1.66	1	6.45	9.49	1	5.30	9.53	0.89	1.37	6.34	9.33
Left lateral	9.37	13.09	0.99	1.06	1.59	1	8.78	13.72	0.99	9.37	13.09	0.91	1.39	8.84	13.75
Prone	8.29	13.37	0.99	1.10	1.87	1	9.44	14.70	0.98	8.28	13.46	0.90	1.75	9.44	14.70
Average	7.89	12.17	0.99	1.12	1.71	1	7.88	12.81	0.99	7.77	12.11	0.89	1.46	7.85	12.75

## Data Availability

Data is unavailable due to privacy or ethical restrictions.
